# Nasal cells as a bronchial cell surrogate for pre-clinical assessment of drug response in cystic fibrosis

**DOI:** 10.3389/fphar.2025.1651122

**Published:** 2025-09-05

**Authors:** Malina Barillaro, Julie Avolio, Sharaniyaa Balachandran, Claire Bartlett, Wan Ip, Hong Ouyang, Wenming Duan, Joseph Zabner, Shaf Keshavjee, Christine Bear, Theo J. Moraes, Tanja Gonska

**Affiliations:** ^1^ Program in Translational Medicine, Hospital for Sick Children, Toronto, ONT, Canada; ^2^ Toronto Lung Transplant Program, Division of Thoracic Surgery, Toronto General Hospital, Toronto, ONT, Canada; ^3^ Department of Internal Medicine, Roy J. and Lucille A. Carver College of Medicine, University of Iowa, Iowa, IA, United States; ^4^ Molecular Medicine, Hospital for Sick Children, Toronto, ONT, Canada; ^5^ Department of Physiology, University of Toronto, Toronto, ON, Canada; ^6^ Division of Respirology, Department of Pediatrics, University of Toronto, Toronto, ON, Canada; ^7^ Division of Gastroenteterology, Hepatology and Nutrition, Department of Pediatrics, University of Toronto, Toronto, ON, Canada

**Keywords:** nasal cells, cystic fibrosis, CFTR modulator, electrophyiology, *in vitro* model

## Abstract

Patient-derived airway cell cultures are used in personalized medicine strategies for people with cystic fibrosis (pwCF) to predict potential clinical response to cystic fibrosis transmembrane conductance regulator (CFTR) modulator drugs. While bronchial epithelial cells from lung explants (HBEx) are the gold standard for CFTR functional measurements, nasal epithelial cells (HNE) are a more practical tissue source resulting in widespread use for preclinical functional platforms. HNE have so far not been rigorously validated against the gold standard for this purpose. In this study, we collected nasal and bronchial cells, and lung explants from pwCF undergoing lung transplantation as well as non-CF controls. Comparative studies in non-CF cells showed that while CFTR-mediated transepithelial currents in HNE underestimated those in HBEx, the magnitude of the CFTR modulator response was similar between CF HNE, brushed HBE (HBEb), and HBEx with significant correlation between matched HNE and HBEb from 16 pwCF. These findings confirm use of HNE as surrogate of bronchial airway for preclinical drug testing with report of drug responses in relation to the tissue-specific non-CF or baseline controls rather than as absolute results. Furthermore, CF centres offering HNE-based drug testing utilize different techniques, challenging the comparison of results between centres. We show how culture media, use of fresh or freeze-thawed cells as well as difference in Ussing technique impact the magnitude of measured CFTR function, which is why we suggest diligence in reporting of these factors when presenting CFTR modulator drug response results.

## Introduction

Cystic fibrosis transmembrane conductance regulator (CFTR) modulator drugs improve CFTR function by targeting defective CFTR protein to improve cellular trafficking and stabilization of channel expression at the apical membrane (corrector drugs) (Van Goor et al., 2011; Veit et al., 2020) as well as promote CFTR channel opening (potentiator drugs) (Van Goor et al., 2009). CFTR modulator drugs have dramatically changed the trajectory of CF disease by significantly improving lung disease and nutrition, as well as reducing sweat chloride concentration as *in vivo* proof of restored CFTR function in people with CF (pwCF) (Ramsey et al., 2011; Wainwright et al., 2015; Keating et al., 2018; Heijerman et al., 2019). Heterogeneity in clinical response ranges from those who respond very well to those who do not respond. This can be explained by differences in CFTR genotypes but has also been observed among pwCF carrying the same CFTR genotypes which can be a result of other genetic factors, individual pharmacokinetics, drug adherence, etc. (Wainwright et al., 2015; Keating et al., 2018). Furthermore, a subset of pwCF are currently ineligible for CFTR modulator therapies as they either a) do not carry gene variants amenable to current CFTR modulator therapy or b) carry rare CFTR gene variants which were not included in clinical trials or secondary functional studies (Raraigh et al., 2022) and are therefore still excluded from these therapies. Addressing these challenges, preclinical platforms using patient-derived airway or intestinal tissues have been established worldwide to help predict potential clinical drug response and to guide/advocate for its clinical use at an individual level (Burgel et al., 2023).

One way to assess potential drug response is by comparing *in vitro* CFTR function at baseline and after drug treatment in patient-derived tissue where CFTR function can be measured as changes in transepithelial electrical currents with pharmacological activation and inhibition. So far, human bronchial epithelial cells derived from lung explants (HBEx) have constituted the gold standard for these measurements (Van Goor et al., 2006; 2009; Keating et al., 2018). Human nasal epithelial cells (HNE) derived from nasal brushes of pwCF are now commonly used as a HBEx/brushed HBE (HBEb) surrogate as they allow relatively non-invasive and repeatable collection. While HNE have been shown to be a good *in vitro* model to predict clinical CFTR modulator response for pwCF (Pranke et al., 2017; Brewington et al., 2018; Anderson et al., 2021; Noel et al., 2022), there is limited understanding of how they compare to the performance of HBE cells. Our group reported that cultured HNE and HBEb showed similar expression levels in over 90% of genes, including *CFTR* (He et al., 2022). However, there has been no direct comparison of CFTR function and drug response assessment between HBEx as gold standard airway model, HBEb, and HNE, leaving a knowledge gap regarding the extent with which CFTR-mediated electrical currents measured in HNE reflect those measured in HBE.

Furthermore, centres offering individual pre-clinical CF drug testing vary in cell culture and CFTR functional measurement techniques, while struggling to find ubiquitously valid cut-off levels of drug response to help guide a more global approach for CFTR modulator use in those with rare CFTR genotypes. Providing this guidance is important as some jurisdictions, including the United States (Durmowicz et al., 2018) and France (Burgel et al., 2023), have programs which allow patients to trial CFTR modulators when certain CFTR functional thresholds are met in *vitro* tests. We aimed to interrogate the quantity and quality of HNE-measured drug responses in comparison to HBEx to support and validate this global approach. Additionally, we evaluated the impact of different cell culturing and measurements techniques on the magnitude of CFTR function measurements to help interpretation of drug response results among different assessment centres.

## Methods

### Patients

PwCF post lung transplantation attending routine clinical follow-up visits for lung rejection surveillance using bronchoscopy were recruited for this study. The study was approved by the Research Ethics Boards of the Hospital for Sick Children (REB# 1000044783) and the Toronto General Hospital (REB#15–8936). All study subjects and guardians of study subjects with insufficient capacity signed consent forms.

### Bronchial explant cultures

Bronchial tissue from non-CF patients and pwCF was collected during lung transplantation and sent to Phil Karp to the Cell Culture Facility at the University of Iowa, thanks to a collaboration between Dr. J Zabner (University of Iowa) and Dr. S Keshavjee (University of Toronto). Bronchial tissue was used to generate HBEx cell cultures, as previously described (Karp et al., 2002). Briefly, cells were expanded in growth media containing bronchial epithelial cell growth media and 2% Ultroser G (USG media). HBEx were seeded onto transwell inserts (Costar 3,470, 6.5 mm diameter, 0.4 μm pore size; Corning) for 2 weeks at air liquid interface (ALI) in Iowa after which they were sent to our lab at the Hospital for Sick Children. Once received, HBEx cultures were incubated in one of four ALI media for 10–14 days before electrophysiological studies: (1) USG media (Karp et al., 2002), (2) UNC media (Randell et al., 2011) (a gift from Scott Randell, UNC), (3) Pneumacult ALI (PC; StemCell Technologies, Vancouver, BC, CA), or (4) Vertex media + USG (VALI) media (Neuberger et al., 2011). The comparisons between HBEx, HBEb and HNE only included HBEx grown in PC ALI media, unless otherwise stated.

### Nasal and bronchial brushing and culturing

At routine surveillance post-transplant bronchoscopy, a bronchoscopic cytology brush was used to brush the bronchial airway lumen proximal to the anastomosis by the attending Respirologist. In the same setting, nasal brushing was performed by a research nurse or Respirologist using a 3-mm diameter cytology brush.

Bronchial and nasal brushes were processed similarly, as described previously for nasal cells (Terrance et al., 2024). Briefly, cells were dissociated from the brush and seeded on a collagen-coated (PureCol, Advanced Biomatrix) flask (T25 for HNE and T75 for HBEb) in expansion media (Pneumacult Ex, StemCell Technologies) with antibiotics and rho kinase inhibitor (5 µM). At passage 2, cells were seeded on collagen-IV coated Transwell inserts (6.5 mm diameter, 0.4 µm pore size, Corning) at a density of 1x10^5^ cells per insert. Once confluent, cells were cultured at an air liquid interface (ALI) with basal differentiation media (PneumaCult ALI, StemCell Technologies) for 14 days before electrophysiological studies.

For cryopreservation, after passaging cells were resuspended in a freezing media consisting of 70% expansion media, 20% FBS (Gibco), and 10% DMSO. Cryovials were transferred to a Nalgene freezing container (“Mr Frosty”) and placed in a −80°C freezer overnight then to liquid Nitrogen storage the following day for long-term storage.

### Electrophysiological studies

Electrophysiological measurements were performed using a circulating Ussing chamber system (EM-CSYS-4; Physiologic Instruments) in open-circuit mode as previously described (Laselva et al., 2021) with symmetrical buffer solutions containing (in mM): 126 NaCl, 24 NaHCO_3_, 2.13 K_2_HPO_4_, 0.38 KH_2_PO_4_, 1 MgSO_4_, 1 CaCl_2_ and 10 glucose. Where indicated, short-circuit measurements were performed with a low chloride buffer in the apical side containing (in mM): 1.2 NaCl, 115 mM Na-gluconate, 1.24 K_2_HPO_4_, 2.4 KH_2_PO_4_, 1 MgCl_2_, 1 CaCl_2_ 25 NaHCO_3_, 10 glucose. Following inhibition of the epithelial sodium channel (ENaC) with amiloride (100 µM, Spectrum Chemical, Gardena, CA), CFTR function was measured using forskolin stimulation (Fsk, 10 μM, Sigma-Aldrich, United States of America) and CFTR_inh_172 inhibition (CFTR_inh_, 10 μM, EMD Millipore Corp. United States of America). CFTR function is presented as difference in calculated transepithelial current where ΔIeq Fsk is indicative of CFTR activation in response to cAMP stimulation and ΔIeq CFTR_inh_ is indictive of current directly attributed to CFTR in response to CFTR inhibition.

### CFTR modulator drug treatment

For drug studies, airway cultures were incubated for 48 h prior to electrophysiological studies with VX-809 (CFTR corrector, Lumacaftor, 3µM, Selleckchem) and/or PTI-428 (CFTR amplifier, nesolicaftor, 10 µM, Proteostasis Therapeutics) or 0.1% DMSO. VX-770 (CFTR potentiator, Ivacaftor, 1 µM, Selleckchem) was added acutely during Ussing chamber experiments.

### Statistical analysis

Individual experimental data are summarized as mean ± SD as they distribute normally. Statistical significance was determined using paired t-test, one-way or two-way ANOVA followed by Tukey’s *post hoc* test, using a significance level of 0.05. Pearson’s correlation was used for correlation analysis. All analyses were performed using GraphPad Prism 10.4.1.

## Results

### Sample description

HNE, HBEb, and HBEx were collected from 29 pwCF, 26 of which carried F508del/F508del. Table 1 lists the individual pwCF with successfully grown HNE, HBEb and HBEx cultures. Although we set out to compare CFTR modulator response in HNE, HBEb and HBEx from each participant, culture failures, initial differences in culture media, and time delays in HNE/HBEb collection of up to 2 years after transplant limited complete individual matching. Comparisons between HNE, HBEb, and HBEx were thus made in an unpaired fashion and included only HBEx differentiated in PneumaCult (PC) ALI media to match the media used for HNE and HBEb. However, for 16 pwCF we have paired data from HNE and HBEb.

**TABLE 1:** Demographics of CF subjects.

Subject	Age at Tx	Gender	Genotype	Nasal cell	Bronchial brush	Bronchial explant
1	19	F	∆F508/∆F508	✓	✓ (non-CF)	✓
2	20	M	∆F508/∆F508	✓	✓	
3	19	F	∆F508/∆F508	✓	✓	
4	26	M	∆F508/∆F508	✓	✓	✓
5	25	F	∆F508/∆F508	✓	✓	✓
6	29	F	∆F508/c.2052dupA	✓	✓	✓
7	40	F	∆F508/∆F508	✓	✓ (non-CF)	✓
8	35	M	∆F508/∆F508	✓	✓	✓
9	18	F	∆F508/∆1507	✓	✓	
10	41	M	∆F508/∆F508		✓	✓
11	27	M	∆F508/∆F508	✓	✓	✓
12	34	M	∆F508/∆F508	✓		✓
13	28	M	∆F508/∆F508	✓	✓	✓
14	28	M	∆F508/∆F508			✓
15	24	F	∆F508/∆F508	✓	✓	✓
16	22	M	∆F508/∆F508	✓	✓	✓
17	38	M	∆F508/∆F508	✓	✓	✓
18	24	M	∆F508/∆F508	✓	✓	✓
19	31	F	G85E/G85E		✓ (non-CF)	
20	25	F	∆F508/∆F508	✓	✓	✓
21	23	M	∆F508/∆F508	✓	✓	✓
22	27	M	∆F508/∆F508	✓	✓	✓
23	36	F	∆F508/∆F508		✓ (non-CF)	✓
24	30	M	∆F508/∆F508			✓
25	25	F	∆F508/∆F508		✓ (non-CF)	✓
26	37	M	∆F508/∆F508		✓	✓
27	24	F	∆F508/∆F508	✓	✓	✓
28	34	F	∆F508/∆F508	✓		✓
29	14	M	M1101K/M1101K	✓		✓

HNE and HBEx were collected from 19 to 16 non-CF subjects, respectively. We received HBEx from non-CF lung transplants and HBEb from bronchoscopies of pwCF post-lung transplantation with accidental bronchial brushings distal to the bronchial anastomosis from the newly transplanted non-CF lung. There was no individual matching between collected non-CF tissues.

### Ieq is reduced in HNE compared to HBEx in non-CF

**FIGURE 1:**
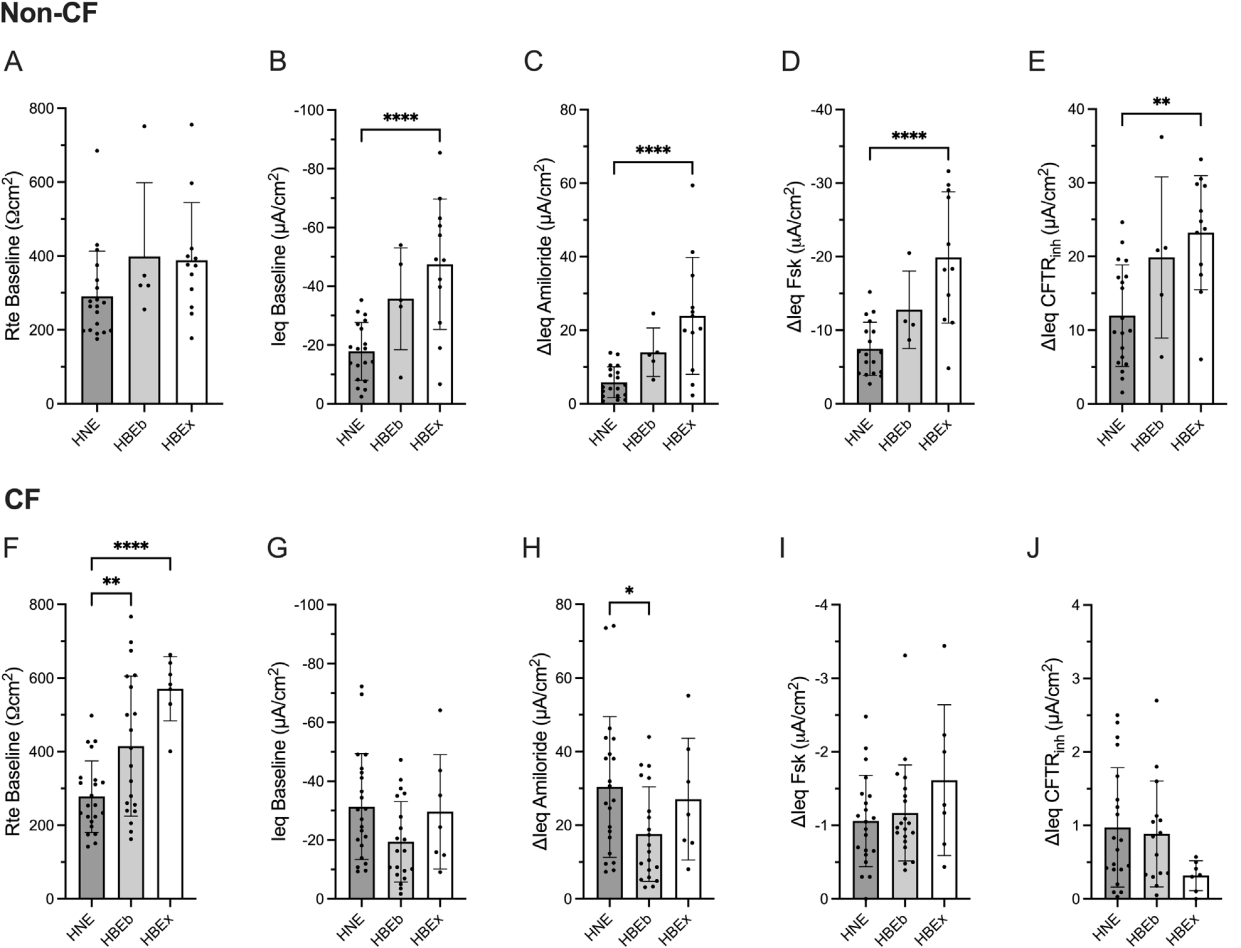
Compared to HBE, non-CF HNE have reduced bioelectric properties, while CF HNE have decreased baseline resistance and increased ENaC activity. Bar graphs with mean ± SD summarizes the transepithelial electrical results from measurements performed in cultures derived from nasal brush (HNE; non-CF, n = 19; CF, n = 22), bronchial brush (HBEb; non-CF, n = 5; CF, n = 20), and bronchial explant (HBEx; non-CF, n = 12; CF, n = 7) grown in Pneumacult ALI media. Each dot represents a single experiment from a single subject, n values provided are indicative of biological replicates. **(A,F)** Baseline transepithelial resistance (Rte baseline, Ωcm^2^), **(B,G)** Baseline transepithelial current (Ieq baseline, µA/cm^2^), and change in transepithelial current with **(C,H)** Amiloride (ΔIeq amiloride, µA/cm^2^), **(D,I)** Forskolin (ΔIeq Fsk, µA/cm^2^) and **(E,J)** CFTR_inh_172 response (ΔIeq CFTR_inh_, µA/cm^2^). Statistical analysis by one way ANOVA: Tukey’s multiple comparison test, *P < 0.05, **P < 0.01, ****P < 0.0001.

Transepithelial electrophysiological properties were evaluated in non-CF airway cultures using open-circuit Ussing chamber studies. While the transepithelial resistance at baseline (Rte baseline, Ωcm^2^) was comparable between HNE, HBEb, and HBEx (Figure 1A), the baseline (Ieq baseline, µA/cm^2^) and amiloride-sensitive current (ΔIeq amiloride) were significantly lower in HNE compared to HBEx (p < 0.0001, Figures 1B,C). Similarly, HNE showed a 2.7- and 1.9-fold reduction in the magnitude of ΔIeq Fsk and ΔIeq CFTR_inh_ as measure of CFTR function, respectively, when compared to HBEx (p < 0.0001 and p = 0.0012, respectively) and were reduced, but not statistically significantly different from HBEb (Figures 1D,E).

### Rte baseline and Ieq amiloride is reduced in HNE compared to HBEx in CF

We next examined transepithelial electrophysiological properties in cultures derived from pwCF. CF HNE showed significantly lower Rte baseline compared to HBEb (p = 0.009) and HBEx (p < 0.0001) (Figure 1F). Additionally, ΔIeq amiloride was significantly greater in HNE compared to HBEb (p = 0.042) but not significantly different from HBEx (Figure 1H), and baseline Ieq was similar between airway cultures (Figure 1G). CFTR activity, assessed as ΔIeq Fsk and ΔIeq CFTR_inh_ was minimal, as expected in CF cells, and similar between airway cultures (Figures 1E,J).

These differences were also observed when including HBEx cultures grown with different ALI media (Supplementary Figure S1).

### The magnitude of responses to CFTR modulator drugs in CF cells is comparable between nasal and bronchial cells

**FIGURE 2:**
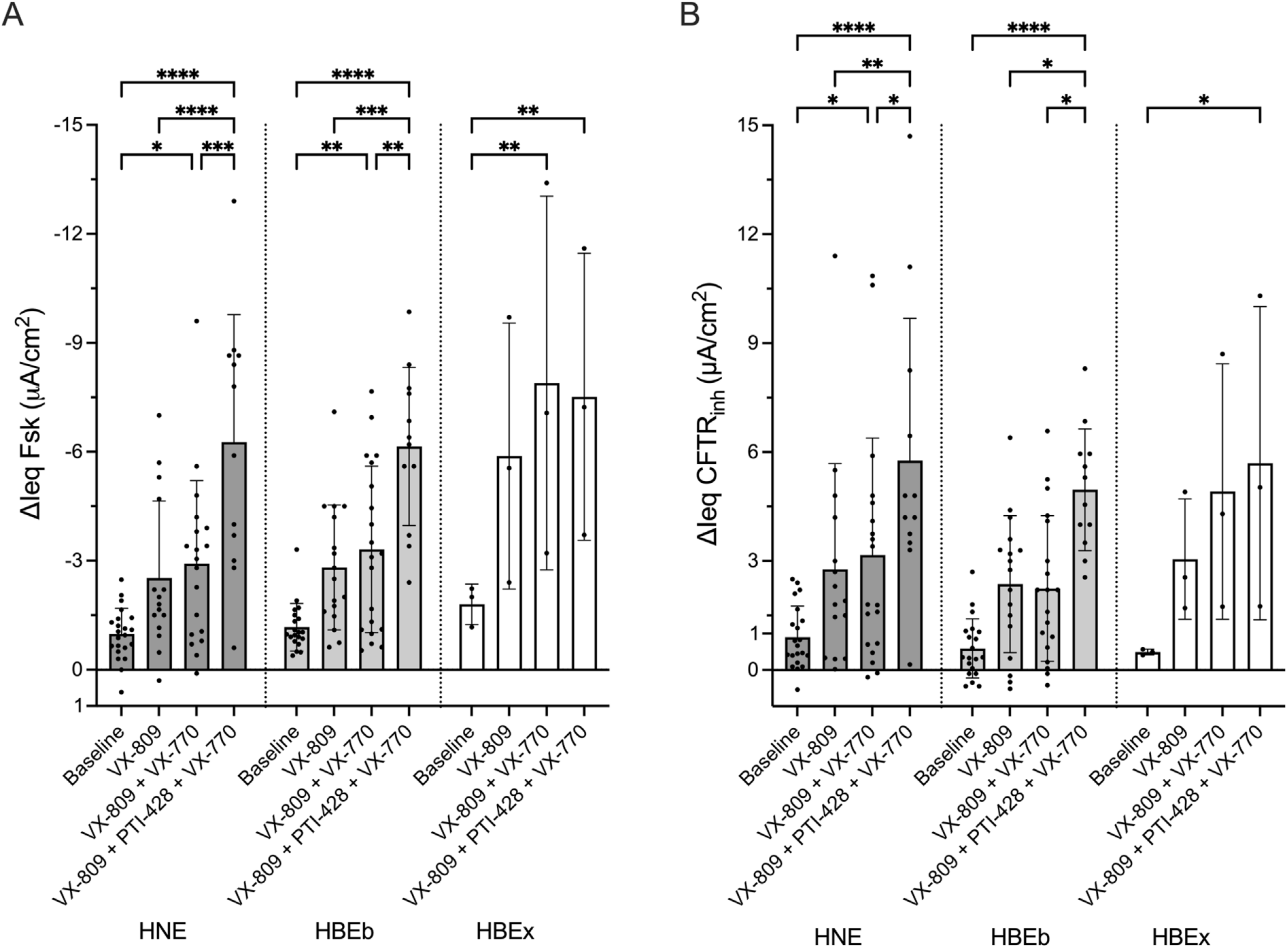
In CF cells, CFTR modulator drug response in HNE is comparable to HBEb and HBEx. Bar graphs with mean ± SD summarizes changes in transepithelial current with **(A)** forskolin-stimulation (ΔIeq Fsk, µA/cm^2^) and **(B)** CFTRinh-172 inhibition (ΔIeq CFTR_inh_, µA/cm^2^) in CF cells derived from nasal brush (HNE, n = 12–19), bronchial brush (HBEb, n = 12–20), and bronchial explant (HBEx, n = 3) cells grown in Pneumacult ALI media. Cells were untreated (baseline) or received 48-h incubation with VX-809, VX-809 with acute VX-770 (VX-809 + VX-770) or VX-809 and PTI-428 with acute VX-770 (VX-809 + PTI-428 + VX-770). Each dot represents a single experiment from a single subject, n values provided are indicative of biological replicates. Statistical analysis by two-way ANOVA: Tukey’s multiple comparison test, *P < 0.05, **P < 0.01, ***P < 0.001, ****P < 0.0001.

Next, we assessed the response to CFTR modulator drugs in HNE, HBEb, and HBEx collected from pwCF. Cells were treated with increasingly potent CFTR modulator combinations (1) VX-809 (Lumacaftor, a CFTR corrector), (2) VX-809 and VX-770 (Ivacaftor, CFTR potentiator), or (3) VX-809, VX-770 and PTI-428 (nesolicaftor, CFTR amplifier). Increasingly efficacious CFTR modulator drug combinations produced stepwise increases in CFTR function measured as ΔIeq Fsk and ΔIeq CFTR_inh_. The functional response to VX-809 alone treatment was only minimal, though associates with increased CFTR protein, as shown previously (Molinski et al., 2017). We observed higher response magnitudes with VX-809+VX-770 and then VX-809+PTI-428+VX-770 (Figures 2A,B). The same patterns were seen when including HBEx grown in different ALI media (Supplementary Figure S2). Importantly, the magnitude of the CFTR response for each drug condition was comparable between HNE, HBEb, and HBEx.

### Interindividual differences in CFTR modulator drug response is similar between nasal and bronchial cells

**FIGURE 3:**
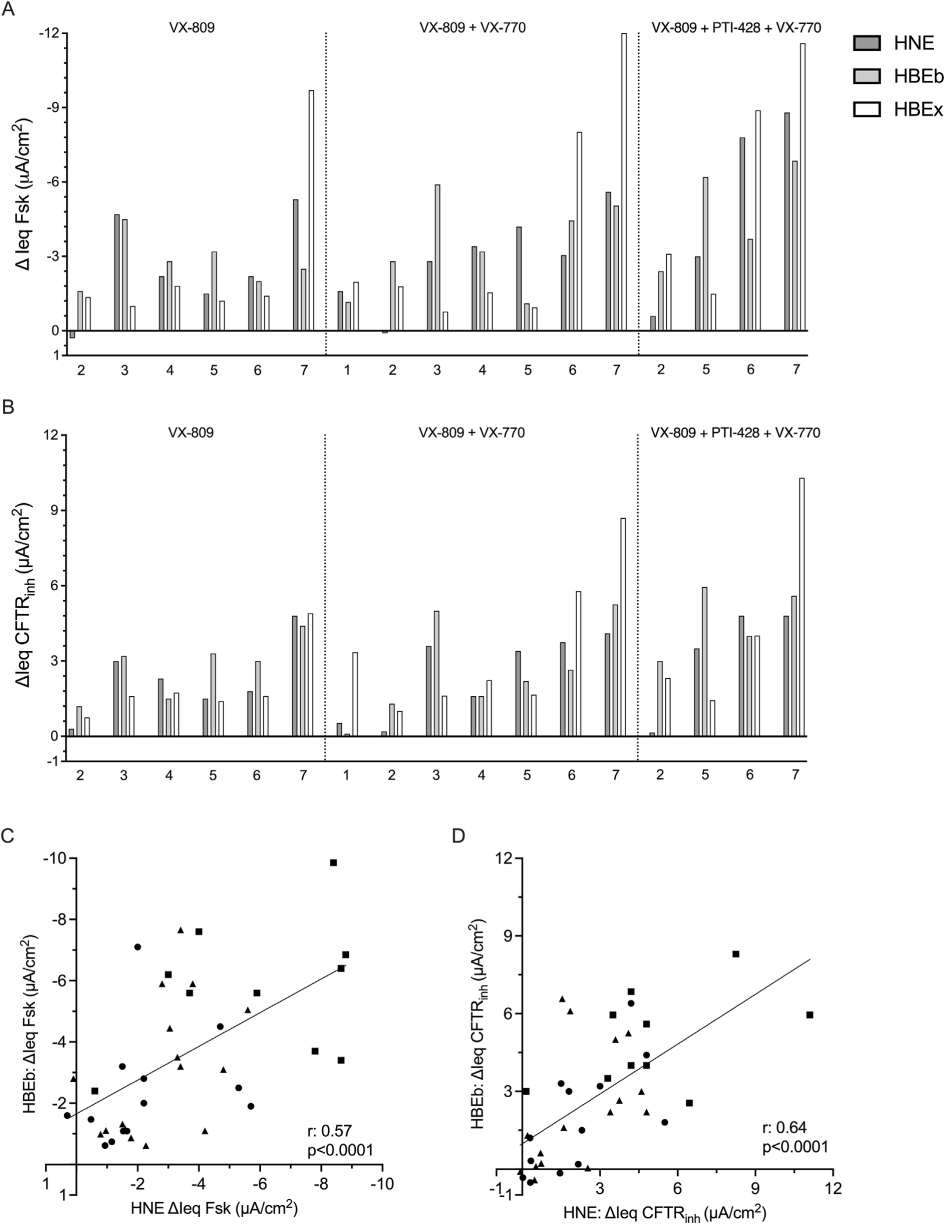
Interindividual variability in response to CFTR modulator drugs is comparable between HNE and HBE. Bar graphs showing the change in transepithelial current with **(A)** forskolin-stimulation (ΔIeq Fsk, µA/cm^2^) and **(B)** CFTRinh-172 inhibition (ΔIeq CFTR_inh_, µA/cm^2^) HNE, HBEb, and HBEx with CFTR modulator drug treatment. Cells received 48-h incubation with VX-809, VX-809 with acute VX-770 (VX-809 + VX-770) or VX-809 and PTI-428 with acute VX-770 (VX-809 + PTI-428 + VX-770). Each bar represents HNE, HBEb, or HBEx from a single individual. Graphs showing correlation of **(C)** ΔIeq Fsk and **(D)** ΔIeq CFTR_inh_ between HBEb (y-axis) and HNE (x-axis) cultures with CFTR modulator treatment (n = 16). Circles: VX-809; Triangles: VX-809 + VX-770; Squares: VX-809 + PTI-428 + VX-770. Statistical analysis by simple linear regression and Pearson’s correlation.

We were also interested in examining whether CFTR modulator drug response in HNE identifies drug responders and non-responders in a similar fashion as HBEb or HBEx. Figures 3A and B show drug responses to the CFTR drug modulator combinations, listed above, in up to 7 individual pwCF using HBEx cultured in any ALI media. Visually we observed good agreement in responses to CFTR modulator drugs between individual pwCF and different airway cells. This agreement suggests that HNE allow for similar interindividual stratification into good and poor response compared to the gold standard HBEx. We observed greater heterogeneity between airway cultures in ΔIeq Fsk compared to ΔIeq CFTR_inh_ (Figures 3A,B). This is likely due to differences cAMP levels prior to forskolin stimulation and thus associated differences in the constitutive currents of the culture. In contrast CFTR_inh_ is applied after maximal CFTR activation and thus captures the sum of constitutive and stimulated CFTR-mediated currents. (Supplementary Figure S3). Lastly, we saw good correlation in both ΔIeq Fsk (r = 0.57, p < 0.0001) and ΔIeq CFTR_inh_ (r = 0.64, p < 0.0001), in CFTR modulator treated matched HNE and HBEb from 16 pwCF (Figures 3C,D). Matched data from HNE and HBEx cultures were only available for six individuals, five of which were cultured in differing media, which likely explains that CFTR function did not correlate between these (data not shown).

### Technical aspects of cell culturing impact CFTR functional read-outs

Since pre-clinical HNE drug testing protocols differ among CF centres worldwide, we wanted to interrogate the impact of some key technical differences on the magnitude of the measured and reported CFTR function.

First, we evaluated the impact of ALI differentiation media on the CFTR functional read-out. We exposed HBEx cultures derived from non-CF and CF lung explants to commonly used ALI differentiation media including Ultroser G (USG), UNC, Pneumacult (PC) and VALI for 10-14 days prior to electrophysiology studies.

**FIGURE 4:**
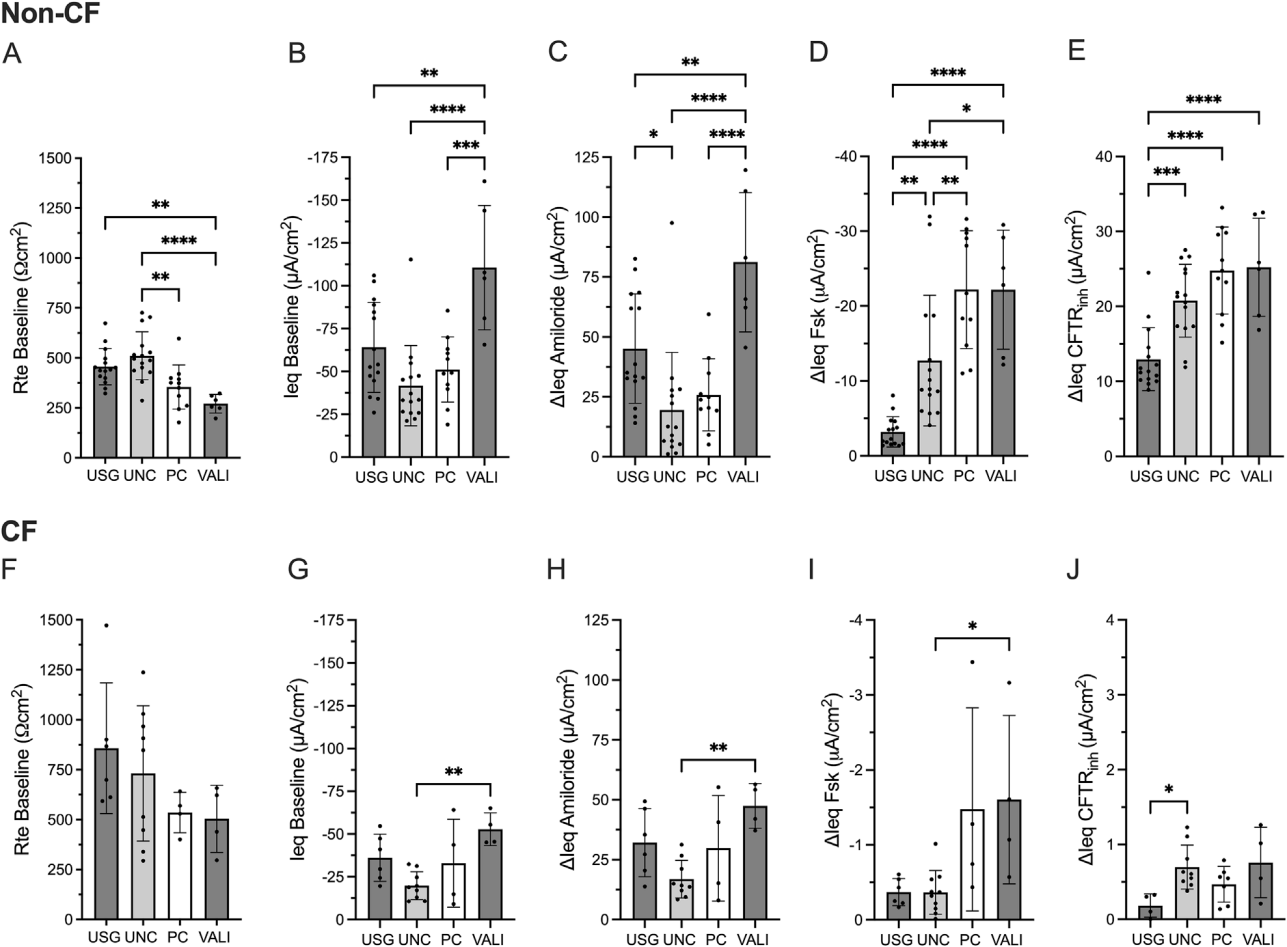
Transepithelial electrical properties in non-CF and CF bronchial explant cultures differ based on ALI media. Bar graphs with mean ± SD summarizes the transepithelial electrical results from measurements performed in non-CF **(A–E)** HBEx in USG (n = 15), UNC (n = 15), PC (n = 11) or VALI (n = 6) ALI media and **(F–J)** CF HBEx in USG (n = 6), UNC (n = 9), PC (n = 4) or VALI (n = 4) ALI media. Each dot represents a single experiment from a single subject, n values provided are indicative of biological replicates. **(A,F)** Baseline transepithelial resistance (Rte baseline, Ωcm^2^), **(B,G)** baseline transepithelial current (Ieq baseline, µA/cm^2^), and change in transepithelial current with **(C,H)** Amiloride (ΔIeq amiloride, µA/cm^2^), **(D,I)** Forskolin (ΔIeq Fsk, µA/cm^2^) and **(E,J)** CFTR_inh_172 response (ΔIeq CFTR_inh_, µA/cm^2^). Statistical analysis by one way ANOVA: Tukey’s multiple comparison test, *P < 0.05, **P < 0.01, ***P < 0.001, ****P < 0.0001.

In non-CF HBEx, transepithelial electrophysiological properties significantly differed between ALI media (Figures 4A–E). Non-CF HBEx grown with USG and UNC ALI media showed higher Rte baseline than PC and VALI HBEx cultures while ΔIeq Amiloride was greatest in VALI followed by USG compared to UNC and PC HBEx cultures. Measured CFTR-mediated currents were significantly larger in PC and VALI HBEx cultures (ΔIeq Fsk of −22.20 ± 7.87 and −22.19 ± 7.93 μA/cm^2^, respectively) compared to UNC (−12.72 ± 8.73 μA/cm^2^, vs. PC p = 0.0067 and vs. VALI p = 0.03) and USG (−3.22 ± 2.02 μA/cm^2^, vs. PC p < 0.0001 and vs. VALI p < 0.0001). Interestingly, HBEx grown in UNC and USG showed greater ΔIeq CFTR_inh_ than ΔIeq Fsk, a consequence of larger constitutive CFTR-mediated currents in comparison to PC and VALI grown cultures. This illustrates that comparison of CFTR function relying solely on ΔIeq Fsk may underestimate total CFTR function depending on ALI media. In HBEx from pwCF, ALI media impacted Rte baseline and ΔIeqs in the same way as non-CF HBEx. Residual measured CFTR currents, though overall low, were also greatest in PC and VALI cultures.

**FIGURE 5:**
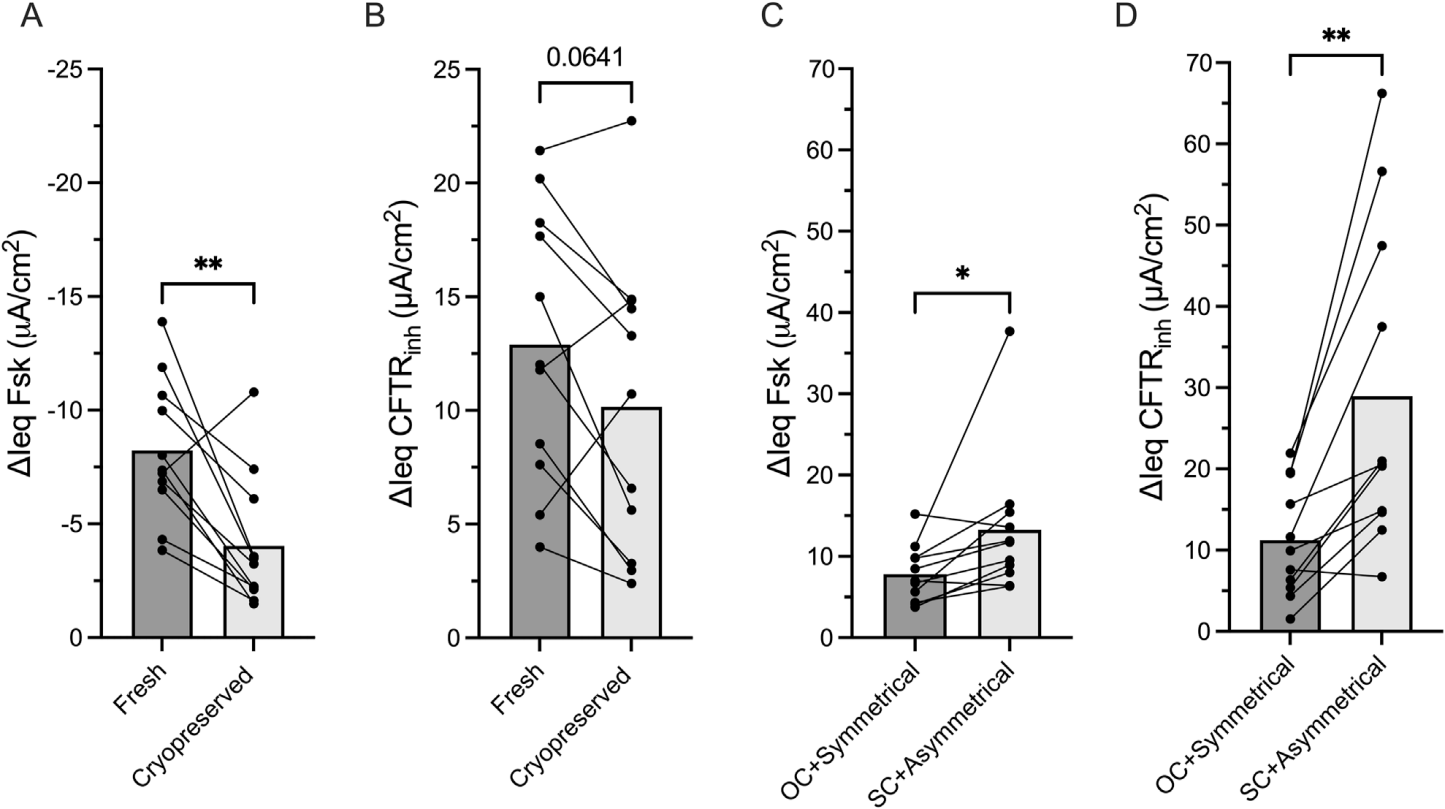
Experimental conditions impact CFTR function measurements Bar graphs with mean ± SD showing the change in transepithelial current with **(A)** forskolin stimulation (ΔIeq Fsk, μA/cm_2_) current and **(B)** CFTRinh-172 inhibition (ΔIeq CFTR_inh_, μA/cm_2_) in paired non-CF nasal cells (HNE) that had never been frozen (fresh) or had been thawed after cryopreservation. **(C)** ΔIeq Fsk and **(D)** ΔIeq CFTR_inh_ in paired non-CF HNE comparing open-circuit and short-circuit Ussing chamber studies. Each dot represents a single experiment from a single subject, n values provided are indicative of biological replicates. Statistical analysis by paired students t-test, *P < 0.05, **P < 0.01.

We also examined the effect of cell cryopreservation on CFTR functional measurements in non-CF HNE. In cryopreserved HNE, ΔIeq Fsk reduced by 50% (p = 0.003), and ΔIeq CFTR_inh_ reduced by 20% (p = 0.064) compared to fresh HNE (Figures 5A,B).

Lastly, we evaluated the impact of commonly used electrophysiological techniques on the magnitude of CFTR functional measurements as most centres measure CFTR function under short-circuit conditions with the application of a basolateral to apical chloride gradient, while a few others use symmetrical chloride and open-circuit conditions. In paired analysis of non-CF HNE, we demonstrated that ΔIeq Fsk and ΔIeq CFTR_inh_ were significant larger under short-circuit conditions with a chloride gradient compared to open-circuit condition with symmetrical chloride (p = 0.04 and p = 0.002, respectively, Figures 5C,D).

## Discussion

In this study, we demonstrate that HNE discriminated between different levels of CFTR modulator responses on an individual and interindividual level in pwCF and performed similarly to HBE. In non-CF cultures, HNE underestimated CFTR function compared to HBE. We also highlight that care needs to be taken when comparing CFTR functional read outs due to significant impact of cell culturing and measurement techniques.

HBEx have laid the foundation for *in vitro* CFTR functional measurements and have been instrumental to assess the efficacy of CFTR modulator drugs (Van Goor et al., 2009; Pranke et al., 2017; Brewington et al., 2018; Keating et al., 2018; Anderson et al., 2021). Since these cells do not suit personalized medicine approaches, HNE are being widely utilized for preclinical drug testing platform building. It has been shown that HNE have the capability to link with *in vivo* outcomes, but there are only few studies directly comparing their performance to HBE. In a comparison study of matched HNE and HBEb, but not HBEx, from ten pwCF with a wide variety in CFTR gene variants, Brewington et al. reported comparable magnitudes of CFTR function when these were treated with VX-809+VX-770 and showed that CFTR function correlated between these two cell types (Brewington et al., 2018). Pranke et al. showed similar VX-809+VX-770-induced changes in CFTR function between HNE and HBEx sampled from 22 pwCF (unmatched) with various CFTR gene variants (Pranke et al., 2017). In this study, we took the comparison one step further trying to overcome limitations of the previous studies. We showed comparable CFTR modulator responses between HNE, HBEb, and HBEx collected from a cohort of 28 pwCF with mainly *F508del/F508del* CFTR variants. While VX-809 produces minimal levels of CFTR rescue, it was the only CFTR corrector available at the time of study. We report small changes in magnitude of CFTR function with VX-809 alone, which are comparable to those measured by Farinha et al. (Farinha et al., 2015). While we showed only changes in functional expression in this manuscript, our group has previously shown that the changes in CFTR function are associated to increase in mature protein expression in Western blot analysis (Molinski et al., 2017). More robust increases in CFTR function were measured with addition of PTI-428, a CFTR amplifier (Giuliano et al., 2018; Dukovski et al., 2020). Additionally we report correlation of CFTR function between 16 subject-matched HNE and HBEb at an individual level. Importantly, in addition to comparing baseline to rescue level, layering of CFTR modulator combinations allowed us to demonstrate that HNE, similarly to HBEb and HBEx, not only discriminated different response levels between individual pwCF (responders and non-responders), but also between different levels of rescued CFTR function within each individual pwCF. This is the prerequisite for using HNE-based pre-clinical platforms to allow ranking drug responses rather than just assessing for yes or no response.

While HNE and HBE cultures have the structural qualities of a mature respiratory epithelia including pseudocolumnar structure, ciliated cells, and mucus-secreting cells following differentiation (Pranke et al., 2017; Brewington et al., 2018) they are distinct tissues with intrinsic differences from function to the transcriptome (Imkamp et al., 2019; Vieira Braga et al., 2019; Deprez et al., 2020; Cho et al., 2021) that can remain even with cell culturing (Rodenburg et al., 2023). In CF HNE and HBE, cell composition was found to differ despite being grown under the same conditions with CF HNE having greater prevalence of MUC5AC positive cells and HBE having more ciliated (β-tubulin-positive) cells (Rodenburg et al., 2023). In RNAseq analysis of these cultures, numerous differentially expressed genes were identified many of which being associated with tissue-specific pathways, i.e., genes in pathways involved in lung development were higher in HBE than HNE (Rodenburg et al., 2023). HNE had reduced expression of *ZO-1*, *E-cadherin*, and tight-junction associated claudins explaining the reduced baseline transepithelial resistance (Rte) compared to HBE found reported here and by others (Brewington et al., 2018; Rodenburg et al., 2023). Importantly, no significant differences in expression of *CFTR* or other apical ion transporters including *ANO1*, *SLC26A9*, and *SLC26A4* were reported (Rodenburg et al., 2023), aligning with previous work from our group showing equivalent expression of *CFTR* and other CF lung disease modifier genes (Corvol et al., 2015) in paired fresh and cultured HNE and HBEx from pwCF (He et al., 2022). Thus, while differences remain, key features impacting CFTR functional measures and lung disease in pwCF are similarly captured in HNE and HBE cultures further validating the use of HNE as a HBE surrogate in this setting.

Non-CF CFTR functional measurements are used as a benchmark when examining CFTR modulator response in CF cultures whether by comparing absolute values or reporting response as a percentage of non-CF. However, there is scarce characterization of ion transport between non-CF HNE and HBE. Here we report HNE have reduced ion transport compared to HBEx in non-CF cultures. This aligns with Pranke et al. who showed reduced, albeit not significant, ΔIeq amiloride, Fsk, and CFTR_inh_ in non-CF HNE compared to HBEx (Pranke et al., 2017). In contrast, the magnitude of CFTR modulator induced CFTR function in cells from pwCF was similar between HNE and HBEx. It remains to be seen whether the tissue specific difference in respect to magnitude of CFTR function may only be revealed at higher levels of CFTR function not captured with the CFTR modulators currently examined. However, at the very least with more potent CFTR functional rescue HNE may underestimate but not inflate the absolute CFTR current of HBE.

We were surprised by the extent to which ALI media and Ussing techniques impact the magnitude of measured CFTR function. Nonetheless, our results are consistent with previous works reporting high CFTR function measurements with PC media (Saint-Criq et al., 2020; Scudieri et al., 2020) which has been explained by greater CFTR RNA and protein expression, specifically when compared to human airway cells grown in UNC ALI media (Saint-Criq et al., 2020). There are several studies demonstrating the impact of ALI media on various airway epithelia properties including prevalence of specific cell types such as ciliated cells (Leung et al., 2020; Luengen et al., 2020; Awatade et al., 2023) or ionocytes (Scudieri et al., 2020), epithelial thickness (Leung et al., 2020), viral response (Awatade et al., 2023; Redman et al., 2024), etc. It has yet to be determined which conditions provide the best representation of *in vivo* CFTR function and CFTR modulator drug response. Banking patient tissues using cryopreservation is a valuable technique allowing for repeat functional studies or testing of new CFTR modulators without resampling. Here we showed that cryopreservation decreases the magnitude of CFTR function in non-CF HNE. This supports previous work by Martinovich et al. who showed decreased CFTR functional measurements in non-CF HBEb with cryopreservation (Martinovich et al., 2017), but challenges findings reporting unchanged CFTR after cryopreservation in CF HNE and HBEb (Martinovich et al., 2017; Kelly et al., 2022). Finally, there have been decades of debate on techniques used in Ussing chamber studies of transepithelial ion transport which may change depending on the research question at hand. While short-circuit with asymmetrical chloride concentrations drive maximal chloride currents across the epithelium (including paracellular spaces), open-circuit with symmetrical chloride concentrations is believed to better reflect the physiological *in vivo* setting. Both techniques are commonly used to report CFTR functional measurements. Here we demonstrate that the magnitude of CFTR function is increased with an apical chloride gradient under short-circuit measurements when compared to symmetrical chloride and open-circuit measurements in matched non-CF HNE. This corresponds with previous work demonstrating the magnitude of CFTR function increased with presence of a chloride gradient in short-circuit (Bratcher et al., 2020). However, chloride and voltage mode may both independently impact CFTR functional measurements and remains to be examined. Cumulatively, with the use of different cell culture and measuring techniques, care need to be taken when comparing CFTR modulator results between different centres.

The main limitation of this study was the unavailability of elexacaftor, texacaftor and ivacaftor (ETI) during the study period. ETI is known as a highly efficient CFTR modulator combination and is widely implemented in clinical therapy. This level of functional correction may have impacted the magnitudes of CFTR drug response in our different cell cultures, widening the difference between HNE and HBEx, as seen in non-CF cultures. However, the window for such experiments was small, and with the introduction of this drug combination, the number of lung transplantation and thus availability of HBEx, has dropped. Additionally, while we set out to compare between HNE, HBEb, and HBEx on an individual level and we successfully cultured all 3 cell types from 7 patients, most HBEx were grown in USG media and given the significant impact of differences in ALI media on CFTR function, we decided against a direct parallel comparison.

In summary, while HNE may underestimate HBE at high levels of CFTR function seen in non-CF, the magnitude of CFTR modulator response in CF HNE correlates to matched HBEb and can discriminate between increments of CFTR rescue as well as stratify good and poor response equal to HBEb and HBEx cultures. Thus, HNE is a reliable and feasible tool for preclinical prediction platforms. In our global endeavor to assign all pwCF effective drug treatments, including those with rare CFTR mutations, collaboration between centres providing these preclinical functional platforms need to be fostered. We suggest that CFTR function should be reported as absolute as well as percent of baseline or non-CF controls grown in the same conditions and details about culture passage, fresh/frozen, media and Ussing conditions should be shared to allow accurate data comparisons and interpretation of results.

## Data Availability

The raw data supporting the conclusions of this article will be made available by the authors, without undue reservation.
