# Supplementary material for: Nasal cells as a bronchial cell surrogate for pre-clinical assessment of drug response in cystic fibrosis
Source: Front Pharmacol. 2025 Sep 5;16:1651122. doi: 10.3389/fphar.2025.1651122 (PMC12446349; doi:10.3389/fphar.2025.1651122)
Supplement: Supplementary file 1 [file Supplementaryfile1.docx]

Supplementary Material

# Supplementary Figures


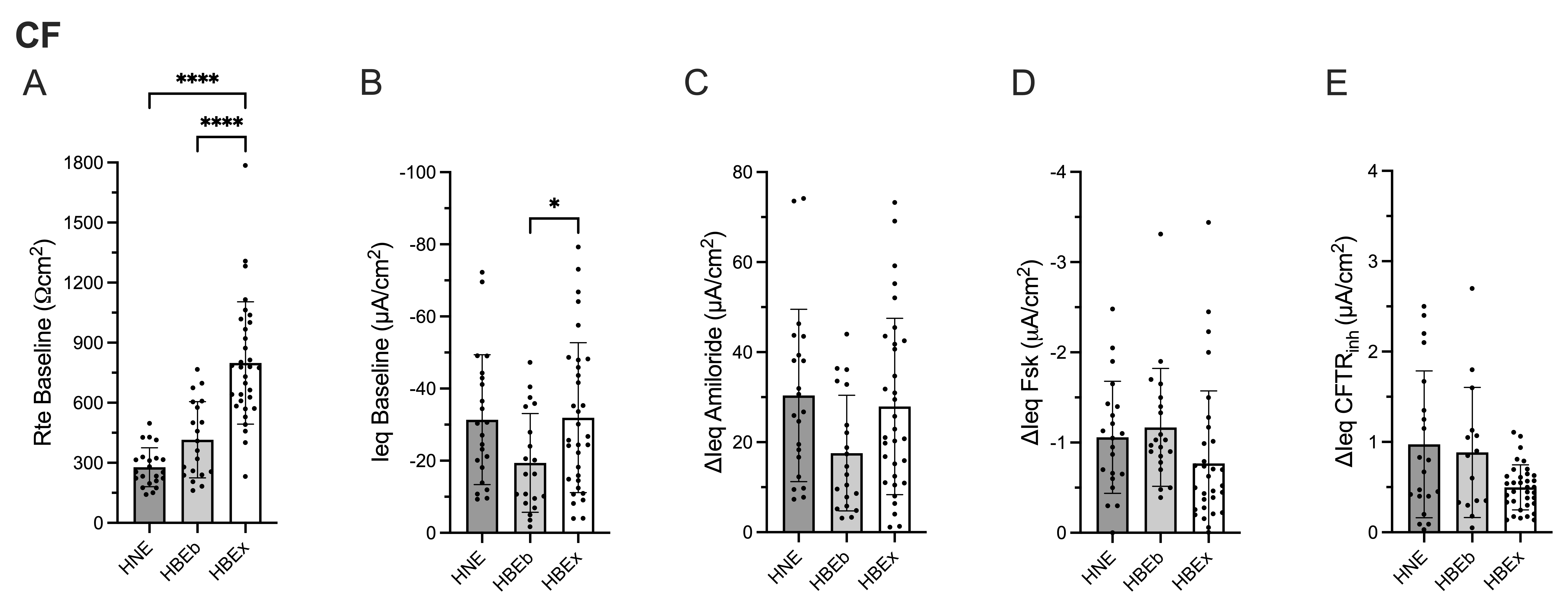


**Supplementary Figure 1.** Bar graphs with mean ± SD summarizes the transepithelial electrical results from measurements performed in cultures derived from nasal brush (HNE, n= 22), and bronchial brush (HBEb, n= 20) grown in Pneumacult ALI media, and bronchial explant (HBEx, n= 25) grown in Pneumacult, UNC, or USG ALI media. Each dot represents a single experiment from a single subject, n values provided are indicative of biological replicates. (**A**) Baseline transepithelial resistance (Rte baseline, Ωcm^2^), (**B**) Baseline transepithelial current (Ieq baseline, µA/cm^2^), and change in transepithelial current with (**C**) Amiloride (ΔIeq amiloride, µA/cm^2^), (**D**) Forskolin (Δ Ieq Fsk, µA/cm^2^) and (**E**) CFTR_inh_172 response (Δ Ieq CFTR_inh_, µA/cm^2^). Statistical analysis by one way ANOVA: Tukey’s multiple comparison test, *P < 0.05, ****P < 0.0001.


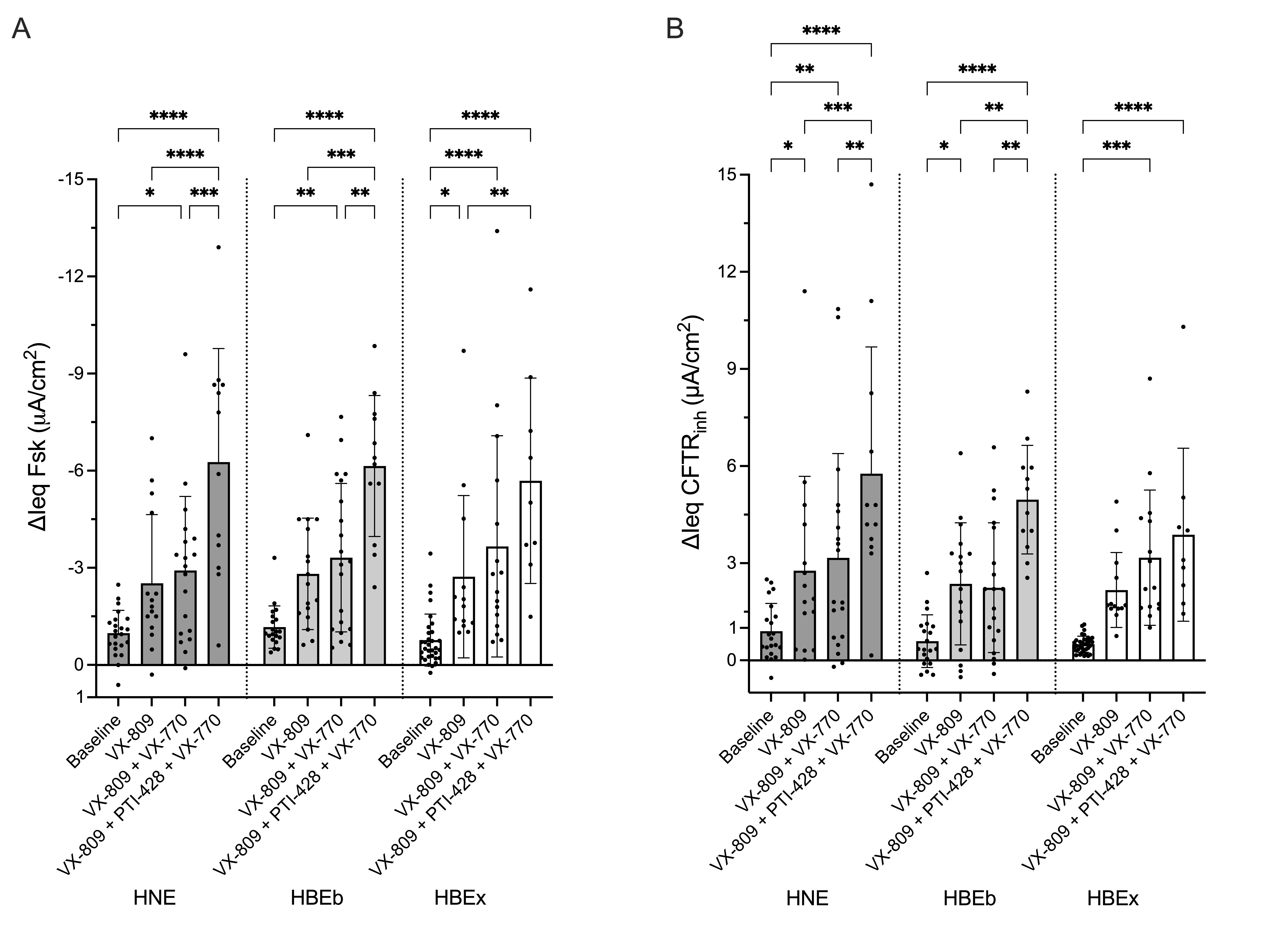


**Supplementary Figure 2.** Bar graphs with mean ± SD summarizes changes in transepithelial current with (**A**) forskolin-stimulation (Δ Ieq Fsk, µA/cm^2^) and (**B**) CFTRinh-172 inhibition (Δ Ieq CFTR_inh_, µA/cm^2^) in CF cells derived from nasal brush (HNE, n=12-19), bronchial brush (HBEb, n=12-20), and bronchial explant (HBEx, n=9-21) cells. Cells were untreated (baseline) or received 48-hour incubation with VX-809, VX-809 with acute VX-770 (VX-809 + VX-770) or VX-809 and PTI-428 with acute VX-770 (VX-809 + PTI-428 + VX-770). Each dot represents a single experiment from a single subject, n values provided are indicative of biological replicates. Statistical analysis by two-way ANOVA: Tukey’s multiple comparison test, *P < 0.05, **P < 0.01, ***P<0.001, ****P < 0.0001.


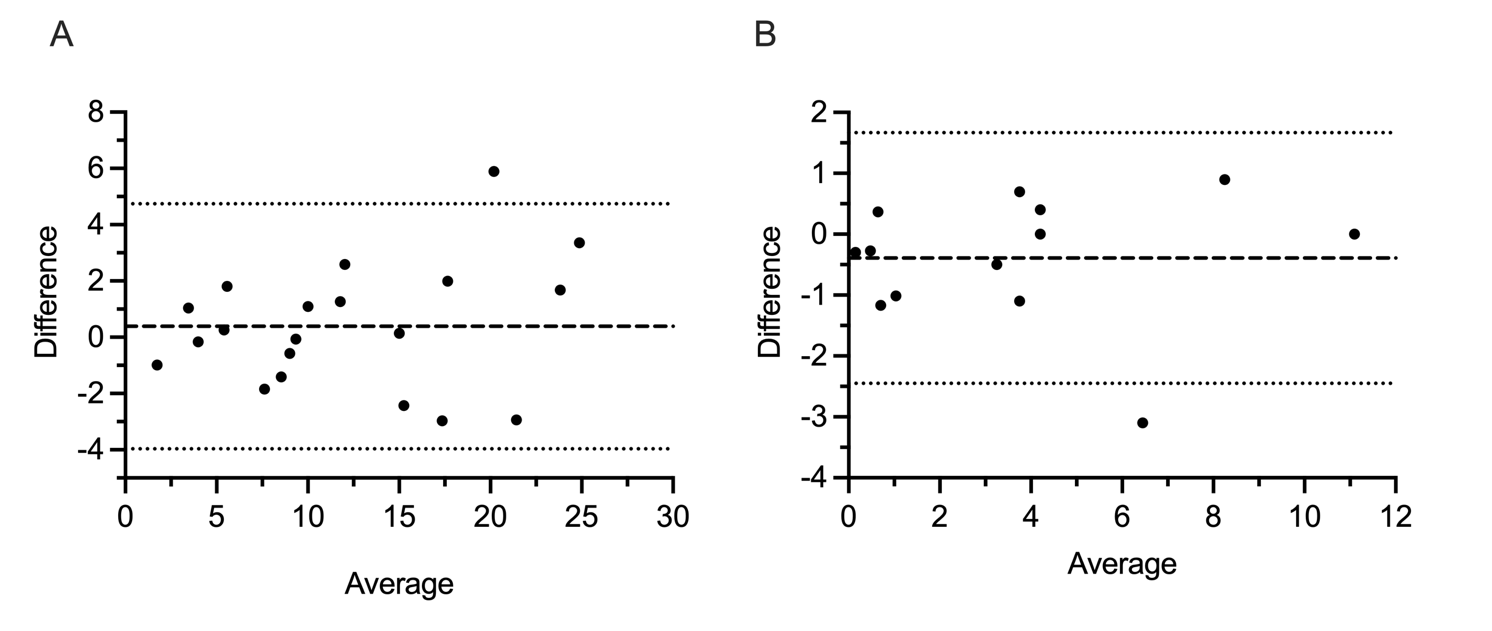


**Supplementary Figure 3.** Bland-Altman plots comparing CFTR_inh_172 response (Δ Ieq CFTR_inh_, µA/cm^2^) against their average in technical replicates of (**A**) non-CF nasal cells (n=18) and (**B**) CF (n=12) nasal cells with CFTR modulator treatment (VX-809+VX-770 or VX-809+PTI-428+VX-770). Dotted line indicates the 95% limits of agreement, and dashed line indicates the bias.
